# Knee varus alters three-dimensional ankle alignment in standing- a study with upright computed tomography

**DOI:** 10.1186/s12891-022-05235-7

**Published:** 2022-04-04

**Authors:** Satoshi Hakukawa, Kazuya Kaneda, Satoshi Oki, Kengo Harato, Yoshitake Yamada, Yasuo Niki, Takeo Nagura, Masaya Nakamura, Masahiro Jinzaki

**Affiliations:** 1 Department of Orthopedic Surgery, Keio University Graduate School of medicine, 35, Shinanomachi, Shinjuku-ku, Tokyo, 160-8582 Japan; 2 Department of Orthopedic Surgery, Keio University School of medicine, Tokyo, Japan; 3 Department of Radiology, Keio University School of medicine, Tokyo, Japan; 4 Clinical Biomechanics, Keio University School of medicine, Tokyo, Japan

**Keywords:** Hindfoot, Weightbearing, Whole legs alignment, Upright computed tomography

## Abstract

**Background:**

For knee osteoarthritis (OA) treatment, it is important to correct the lower limb alignment including the foot. However, in the upright position, lower limb alignment is generally assessed from the body surface or radiographs, and it is a challenge to capture the exact characteristics of three-dimensional lower limb alignment. The purpose of the study was to measure lower limb alignment in patients with knee OA using upright computed tomography (CT) and radiography, and to identify features of knee joint deformity.

**Methods:**

A total of 45 limbs in 25 patients with knee OA were enrolled. The subjects underwent both upright CT and radiography for the whole lower limb in the standing position. The joint angles were calculated on both images. The degree of knee OA was classified according to Kellgren-Lawrence (KL) grade by referring to radiography, which is mainly based on the degree of articular cartilage loss and severity of osteophytes, and the characteristics or correlation between knee and ankle joint in each group was investigated.

**Results:**

In KL-I, there was an association between varus of the knee joint and internal rotation of the talocrural joint (*r* = 0.76, *P* < 0.05). In KL-II, there was an association between varus of the knee joint and eversion of the subtalar joint (*r* = 0.63, *P* < 0.05) and talocrural joint (*r* = − 0.65, *P* < 0.05). In KL-III, there was an association between varus of the knee joint and internal rotation of the subtalar joint (*r* = − 0.62, *P* < 0.05), and in KL-IV, there was an association between varus of the knee joint and internal rotation of the subtalar joint (*r* = − 0.58, *P* < 0.05).

**Conclusions:**

The lower limb alignment of patients with knee OA in the standing position was found that as knee OA worsened, it became apparent that compensatory knee joint alignment depended on the ankle joint rather than the subtalar joint. The results may help in the rehabilitation of patients with knee OA, since the ankle joint alignment has a significant impact on the knee joint during coarse movements involving load.

## Background

Osteoarthritis (OA) is a musculoskeletal disease that affects approximately 300 million people worldwide, with knee OA being the most common OA disease [1]. The main pathology of knee OA is three-dimensional (3D) deformity of the lower limb, and the main changes occur on the coronal plane as varus or valgus deformities [2]. Andriacchi et al. [3] state that normal cartilage depends on three factors: biology (cellular metabolism, inflammation), functional mechanics, and structure. In the cases of knee OA, it is known that the alignment also changes at the ankle joint. The changes occurring in the hindfoot are thought to be compensatory changes to restore the neutral hip-knee-ankle coronal alignment [4]. However, 3D changes in the lower extremity of each joint remain unclear since the evaluation of the lower limb joints is usually based on two-dimensional (2D) standing radiographs. In particular, due to this technical limitation of 2D radiography, the evaluation of hindfoot alignment was limited on the coronal plane.

To overcome this limitation, computed tomography (CT) has been used to evaluate the 3D alignment of the hindfoot and ankle. In the study by Kimura et al. [5], simulated weight was applied in the supine position, but the effect of gravity and lower limb muscle were neglected. Recently, upright cone-beam CT scanners that can acquire images of the foot and ankle in standing position have been used [6]. Collan et al. [7] used cone-beam CT to evaluate the alignment of the big toe under weightbearing condition. In addition, Carrino et al. [8] verified the accuracy of cone-beam CT and showed its usefulness. However, cone-beam CT can only acquire a partial image of the lower limbs, and since it is taken in an unnatural standing position, there is a limitation that the lower limbs cannot be evaluated comprehensively. Therefore, the 3D alignment change occurring under weightbearing between the OA knee and the hindfoot has not been clarified.

Evaluation of the hindfoot alignment in knee OA patients is an important issue when we treat knee OA using orthosis. Lateral wedge insole has been used in varus knee OA to modify either lower limb alignment or knee joint loads during gait [9–11]. The lateral wedge insole is considered to affect the coronal plane to realign the lower limb and mechanical axis. Since deformity of knee OA involves 3D change between femur and tibia, re-alignment of the lower limb only on the coronal plane may result in the insufficient or adverse effect of the insole [12]. Akasaki et al. analyzed the alignment of standing radiographs of patients with knee OA while wearing a lateral wedge insole and reported that the joint line convergence angle of the knee joint improved by about 3 degrees at a 20-degree inclination [13]. However, a three-dimensional evaluation including changes in the horizontal plane has not been conducted. To obtain better clinical results with the insole, it will be beneficial if we are able to evaluate 3D alignment change of the hindfoot in knee OA patients. Especially in gait, patients with knee OA are said to be characterized by external rotation of the lower leg during loading [14], and it is important to make a three-dimensional assessment of the relationship between the lower leg and foot kinetic chain during loading in patients with knee OA.

The purpose of this study was to investigate the relationship between the knee and hindfoot alignment in patients with knee OA by assessing the lower limb alignment using upright CT. We hypothesized that varus deformity of the knee joint causes changes in hindfoot alignment not only in the coronal plane but also in the sagittal and axial planes.

## Methods

### Subjects

48 limbs of 25 patients (21 women, 4 men) with medial knee OA participated in this study. However, three of these limbs were lower limbs with a history of surgery and were excluded because of their potential to influence the analysis, and a total of 45 limbs were enrolled. Patients with valgus knee, a history of foot or knee trauma, and inflammatory disease such as rheumatoid arthritis, were excluded. The mean (± standard deviation) age, body weight, and body mass index (BMI) of the participants were 69.8 ± 9.0 years, 58.4 ± 13.0 kg, and 24.2 ± 5.1 kg/m^2^, respectively.

### Image acquisition

**Fig. 1:**
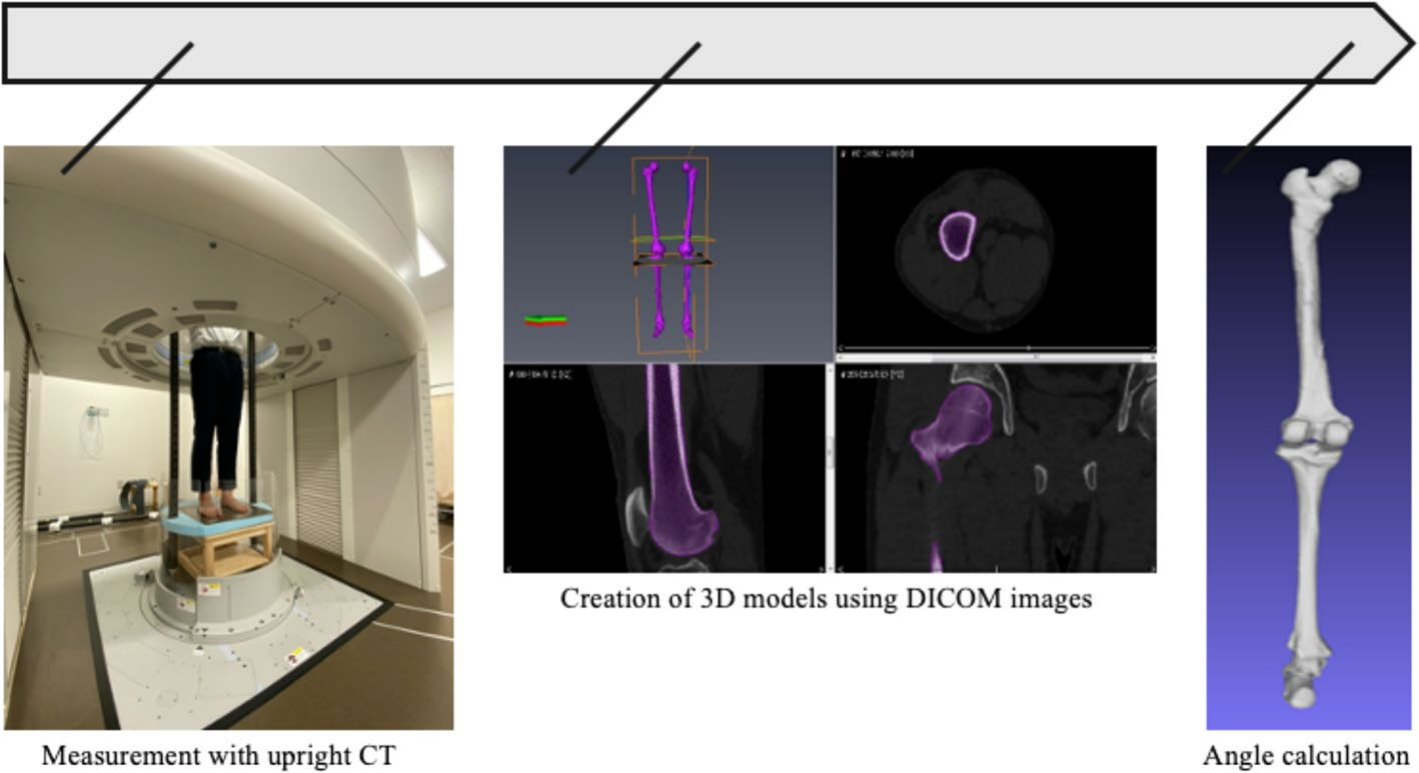
Flow diagram from measurement with upright computed tomography (CT) to angle calculation. The CT images were acquired from the distal femur to the whole foot using a 320 detector row upright CT scanner (prototype TSX-401R; Canon Medical Systems, Otawara, Japan). The condition of each weightbearing stance was measured using a pressure mat (BIG-MAT; NITTA Corporation, Osaka, Japan) and pressure calculation system (FootMat; Tekscan, South Boston, MA, USA)

The CT images were acquired total length of lower limb to the entire foot using the 320-row upright CT scanner (proto-type TSX-401R; Canon Medical Systems, Otawara, Japan) (Fig. 1) [15] and in view of the radiation exposure of the subjects [16]. The CT examinations were performed using the following parameters: peak tube voltage, 100 kV; tube current, 10 to 350 mA (using a noise index of 15 for a slice thickness of 5 mm); rotation speed, 0.5 s; and slice thickness, 0.5 mm. In an upright CT scanner, all participants stood in a relaxed position with their bare feet shoulder-width apart. The condition of weightbearing stance was measured using a pressure mat (BIG-MAT; NITTA Corporation, Osaka, Japan) and pressure calculation system (FootMat; Tekscan, South Boston, MA, USA) because we ensure that the weightbearing is evenly distributed on both sides of foot. The patient’s posture was such that the weight of the patient was placed on both feet as evenly as possible, with the toes facing forward. The CT data were accumulated using the Digital Imaging and Communication in Medicine (DICOM) data format.

### Coordinate system and definition of the joint angles

**Fig. 2:**
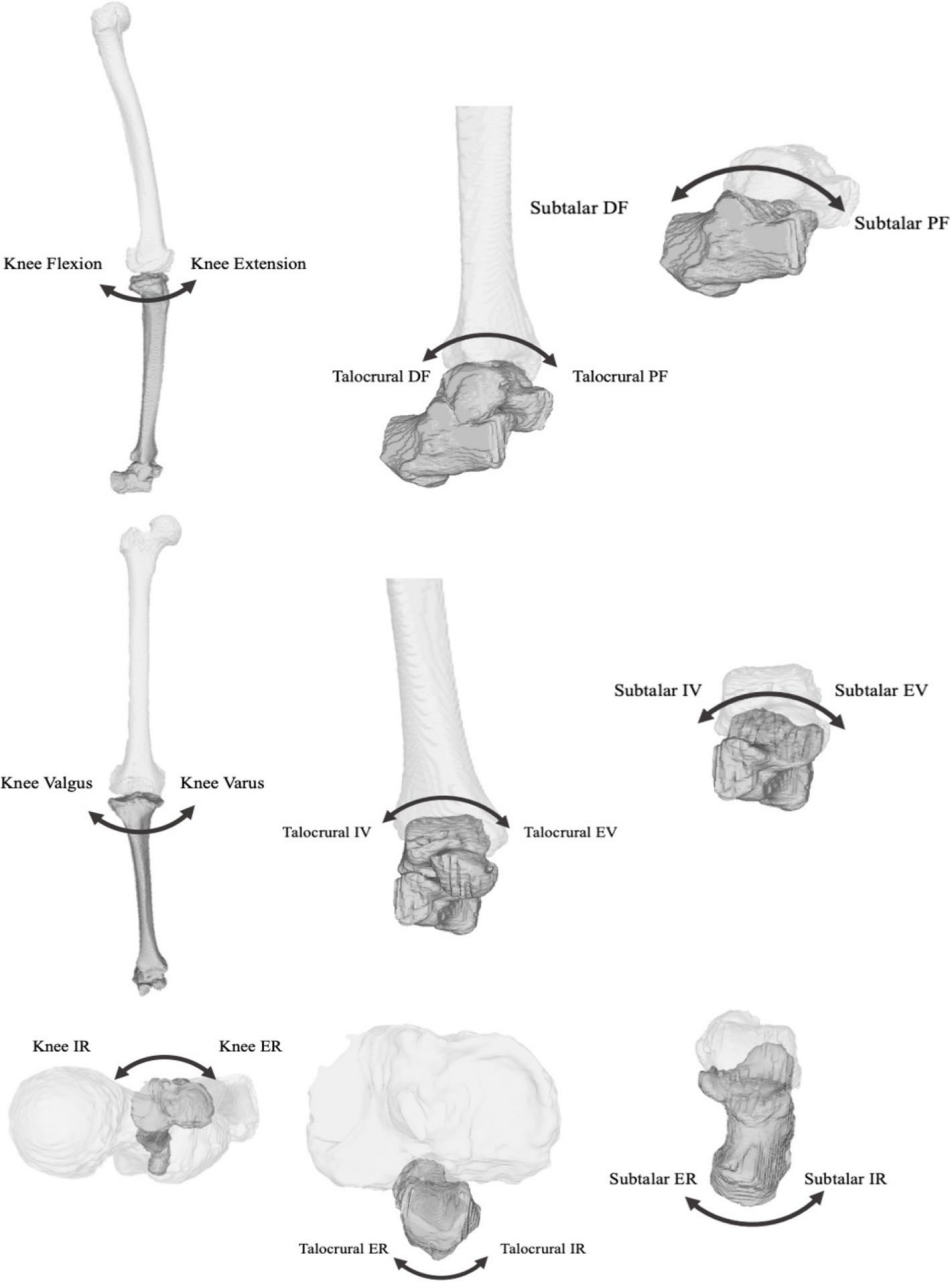
Three-dimensional angles of the knee joint, the talocrural joint and the subtalar joint were defined. As the knee joint angle increased/decreased, the sagittal, coronal, and horizontal planes were defined as extension/flexion, varus/valgus, and tibial internal/external rotation; the talocrural joint angle was defined as dorsiflexion/plantarflexion, inversion/eversion, and internal/external rotation; and the subtalar joint angle was defined as dorsiflexion/ plantarflexion, inversion/eversion, and internal/external rotation

Three-dimensional surface data of the femur, tibia, talus, and calcaneus were extracted from the CT DICOM data using 3D visualization software (AVIZO 9.0.1; Thermo Fisher Scientific, Tokyo, Japan). We modified and used the coordinate system of the femur and tibia using the method defined by Sato et al. [17] and the International Society of Biomechanics [18]. For the femur, the coordinate points consisting of the origin coincident with the hip centre of rotation, the medial femoral epicondyle and the lateral epicondyle were used, and for the tibia, the coordinate points of the medial and lateral center of the upper articular surface and the center of the lower articular surface were used. The coordinate systems of the talus and the calcaneus were defined using the method described by Gutekunst et al. [19]. For the calcaneus, the coordinates of four points on the medial and lateral edges of the posterior surface of the calcaneal tuberosity were used, and for the talus, the coordinates of four points on the corners of the talar pulley surface were used. We used the Euler/Cardan angles representing three sequential rotations about the anatomical axis of the proximal bone to describe the bone-to-bone rotations; the tibia relative to the femur, the talus relative to the tibia, and the calcaneus relative to the talus, around each axis as to define 3D joint angle about the knee, talocrural joint and subtalar joint, respectively. As the knee joint angle increased/decreased, the sagittal, coronal, and horizontal planes were defined as extension/flexion, varus/valgus, and tibial internal/external rotation; the talocrural joint angle was defined as dorsiflexion/plantarflexion, inversion/eversion, and internal/external rotation; and the subtalar joint angle was defined as dorsiflexion/ plantarflexion, inversion/eversion, and internal/external rotation (Fig. 2).

### Relation between the alignment of the knee joint and talocrural or subtalar joints

Grades of knee OA were classified into four groups by the Kellgren-Lawrence (KL) classification [20], which is mainly based on the degree of articular cartilage loss and severity of osteophytes, based on the evaluation by an orthopedic surgeon with 10 years of clinical experience in knee OA treatment. The characteristics of lower extremity alignment between each group were investigated. The calculated joint angles were tested for normality using the Shapiro-Wilk test, and those with normality were examined for significant differences using one-way analysis of variance and those without normality using the Kruskal-Wallis test. Furthermore, items that were significantly different were subjected to multiple comparisons using the Bonferroni method.

In order to clarify the characteristics of alignment in each grade, Pearson’s correlation coefficients were used to determine the relationship between the alignment of the knee joint, talocrural joint and subtalar joint during standing and Spearman’s rank correlation coefficients were used to determine the relationship between the alignment of the knee joint, talocrural joint and subtalar joint during resting in each group, with Pearson’s correlation coefficients for those that were normal and Spearman’s rank correlation coefficients for those that were not normal. The study was conducted. All levels of significance in the statistical analysis of this study were set at less than 5%. The intra- and inter-observer reliabilities for the for the three-dimensional joint angles were assessed by calculating intraclass correlation coefficient (ICC) based on 12 randomly selected lower limbs. The measurements were made blind by one orthopedic surgeon and one physical therapist (ICC model 2,1) and repeated after a 3-month interval by one physical therapist (ICC model 1,1). After the reliabilities were determined to be acceptable according to the previous report [21], three-dimensional joint angles for all 45 lower limbs were assessed by a single physical therapist.

## Results

### Patients’ demographics

The patients OA grades were classified by measuring from radiography as follows; KL-I = 11 limbs, KL-II = 11 limbs, KL-III = 11 limbs, and KL-IV =12 limbs. The femorotibial angle (FTA) by standing radiography was 174.5 ± 1.5 degrees with KL-I, 176.6 ± 3.1 degrees with KL-II, 179.6 degrees ±4.3 degrees with KL-III, and 187.7 ± 6.2 degrees with KL-IV.

### 3D joint angles in standing

The strength of agreement of the intra- and inter-observer correlation coefficients for the three-dimensional joint angles was almost perfect or substantial (Table 1). The knee flexion, varus, internal rotation of the talocrural joint, and dorsiflexion of the subtalar joint were ‘Almost Perfect’. The internal rotation of the knee joint, dorsiflexion of the talocrural joint, varus, and dorsiflexion of the subtalar joint were ‘Substantial’. These results confirmed that the measurements of the three-dimensional joint angles were highly reproducible.

**Table 1:** Strength of agreement of the intra- and inter-observer correlation coefficients were examined

Joint		ICC model 1,1 Number (95% CI)	SA	ICC model 2,1 Number (95% CI)	SA
Knee	Flexion	0.90 (0.69–0.97)	Almost Perfect	0.92 (0.76–0.98)	Almost Perfect
	Varus	0.94 (0.81–0.98)	Almost Perfect	0.88 (0.60–0.96)	Almost Perfect
	IR	0.77 (0.21–0.94)	Substantial	0.80 (0.45–0.94)	Almost Perfect
Subtalar	DF	0.71 (0.47–0.85)	Substantial	0.93 (0.79–0.98)	Almost Perfect
	IV	0.89 (0.66–0.96)	Almost Perfect	0.88 (0.62–0.96)	Almost Perfect
	IR	0.83 (0.43–0.94)	Almost Perfect	0.81 (0.45–0.94)	Almost Perfect
Talocrural	DF	0.85 (0.67–0.93)	Almost Perfect	0.67 (0.20–0.79)	Substantial
	IV	0.70 (0.24–0.91)	Substantial	0.78 (0.23–0.94)	Substantial
	IR	0.71 (0.14–0.92)	Substantial	0.79 (0.26–0.94)	Substantial

*SA* Strength of Agreement, *IR* Internal rotation, *DF* Dorsiflexion, *IV* Inversion, *IR* Internal rotation

The measurements of the three-dimensional joint angles were highly reproducible

Table 2 showed 3D joint angles (knee, talocrural and subtalar joints) in upright standing. Multiple comparisons revealed that flexion and varus angles of the knee joint increased as the OA grade increased. The tibial external rotation decreased as the knee OA grade increased, and there were significant differences between KL-I and III, or I and IV. Additionally, the dorsiflexion angle of the talocrural joint decreased as the knee OA grade increased, and there were significant differences between KL-I and III, or I and IV.

**Table 2:** Comparison of knee joint, talocrural joint, and subtalar joint in each grade of knee OA

	Plane	KL-I	KL-II	KL-III	KL-IV	*P* value
Knee Joint	Sagittal	−2.32 ± 3.06	1.48 ± 2.52	7.49 ± 7.97	10.22 ± 4.32	.0001**
	Coronal	2.78 ± 2.55	5.26 ± 3.43	8.15 ± 5.17	12.66 ± 5.55	.0001**
	Axial	11.56 ± 3.29	8.10 ± 5.04	3.60 ± 5.77	4.24 ± 8.65	.012*
Talocrural Joint	Sagittal	31.95 ± 7.91	26.86 ± 10.76	19.23 ± 8.89	23.26 ± 9.49	.018*
	Coronal	9.86 ± 7.11	10.19 ± 7.42	7.01 ± 4.51	6.78 ± 5.45	.392
	Axial	5.32 ± 8.98	4.40 ± 14.31	6.65 ± 7.16	10.26 ± 11.31	.574
Subtalar Joint	Sagittal	32.49 ± 8.05	33.32 ± 10.36	27.77 ± 9.66	31.20 ± 9.37	.516
	Coronal	−5.34 ± 5.13	− 6.94 ± 6.69	−7.96 ± 9.23	−6.09 ± 12.55	.875
	Axial	−23.40 ± 3.88	−22.88 ± 3.80	−25.05 ± 6.66	12.66 ± 5.55	.118

KL-I; Slight osteophyte or subchondral osteosclerosis without narrowing of the articular cleft

KL-II; Narrowing of the articular cleft (0–25%)

KL-III; Narrowing of the articular cleft (25–75%), obvious formation of osteophytes, and sclerosis of the subchondral bone

KL-IV; Narrowing of the articular cleft (over 75%), and significant bone changes

Data are presented as one-way analysis of variance (*P* value)

* Indicates significance at *P* < 0.05

** Indicates significance at *P* < 0.005

The angle of the knee joint differed depending on the KL-grade. The angle of the talocrural joint in the sagittal plane differed depending on the KL-grade. There were no differences in the movements of other joints

### Correlation between the knee joint angles and ankle joints angles on each plane

**Fig. 3:**
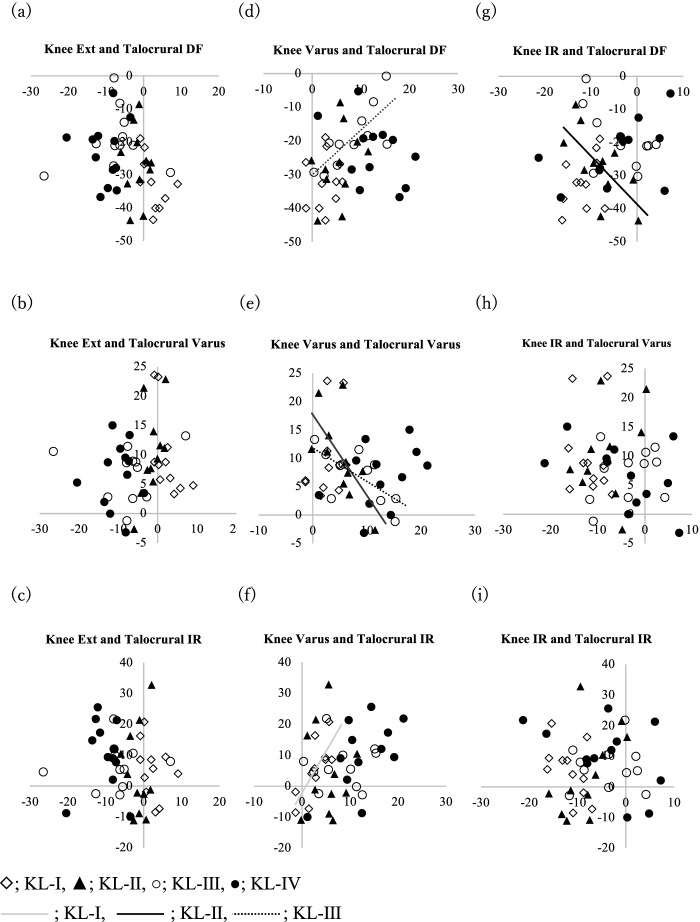
The relationship between the knee and talocrural joint is shown in a scatter plot. The horizontal axis is the knee joint angle, and the vertical axis is the talocrural joint angle (degree). Trendlines were filled in for items with significant differences between the knee and talocrural joint

Table 3 and Fig. 3 (a-i) showed the correlation between knee joint angle and talocrural joint angle on each plane. There were no correlations between sagittal and axial plane joint angles between the knee joint and the talocrural joint. On the coronal plane, significant correlations were found between knee joint varus angle and talocrural inversion angle in all lower limbs (*r = − 0.337, P < 0.05*), the limbs with KL-II knee OA (*r = − 0.65, P < 0.05*), and KL-III knee OA (*r = − 0.67, P < 0.05*) (Fig. 3-e). On the different plane, significant correlations were found between knee joint varus angle and talocrural dorsiflexion angle in the limbs with KL-III knee OA (*r = 0.77, P < 0.05*) (Fig. 3-d), and knee joint varus angle and talocrural internal rotational angle in the limbs with KL-I knee OA (*r = 0.76, P < 0.05*) (Fig. 3-f). Moreover, significant correlations were also found between knee joint tibial internal rotation angle and talocrural dorsiflexion angle in the limbs with KL-II knee OA (*r = − 0.68, P < 0.05*) (Fig. 3-g).

**Table 3:** Correlation between the knee joint and talocrural joint in each grade of knee OA

Joint Plane			Correlation coefficient (*P* value)				
Knee	Talocrural	Chart	All	KL-I	KL-II	KL-III	KL-IV
Sagittal	Sagittal	a)	−0.051	− 0.536	− 0.012	0.120	0.008
			(0.350)	(0.090)	(0.972)	(0.726)	(0.980)
	Coronal	b)	−0.193	0.587	− 0.570	− 0.079	− 0.076
			(0.203)	(0.057)	(0.067)	(0.818)	(0.813)
	Axial	c)	0.134	0.337	−0.032	−0.152	0.022
			(0.381)	(0.311)	(0.925)	(0.655)	(0.945)
Coronal	Sagittal	d)	0.311	0.133	0.350	0.774**	0.447
			(0.125)	(0.696)	(0.292)	(0.005)	(0.145)
	Coronal	e)	−0.337*	− 0.359	− 0.659*	− 0.676*	− 0.368
			(0.032)	(0.279)	(0.027)	(0.022)	(0.240)
	Axial	f)	−0.154	0.760**	−0.007	0.005	0.566
			(0.210)	(0.007)	(0.983)	(0.358)	(0.055)
Axial	Sagittal	g)	0.045	0.404	−0.685*	−0.572	0.101
			(0.229)	(0.217)	(0.020)	(0.066)	(0.496)
	Coronal	h)	0.174	−0.140	0.227	0.307	0.490
			(0.280)	(0.681)	(0.409)	(0.358)	(0.106)
	Axial	i)	−0.052	−0.478	0.549	0.047	0.454
			(0.999)	(0.137)	(0.080)	(0.891)	(0.138)

* Indicates significance at *P* < 0.05

** Indicates significance at *P* < 0.01

Pearson’s correlation coefficients and Spearman’s rank correlation coefficients were used to determine the relationship between the alignment of the knee joint and the talocrural joint. In KL-I, the angle of the coronal plane of the knee joint and the angle of the coronal plane of the talocrural joint were correlated. In KL-II, there were correlations between the angle of the axial plane of the knee joint and the angle of the sagittal plane of the talocrural joint, and the angle of the coronal plane of the knee joint and the angle of the coronal plane of the talocrural joint. In KL-III, the angle of the coronal plane of the knee joint and the angle of the sagittal and coronal plane of the talocrural joint were correlated. In KL-IV, there was no correlation between the knee and talocrural joint

**Fig. 4:**
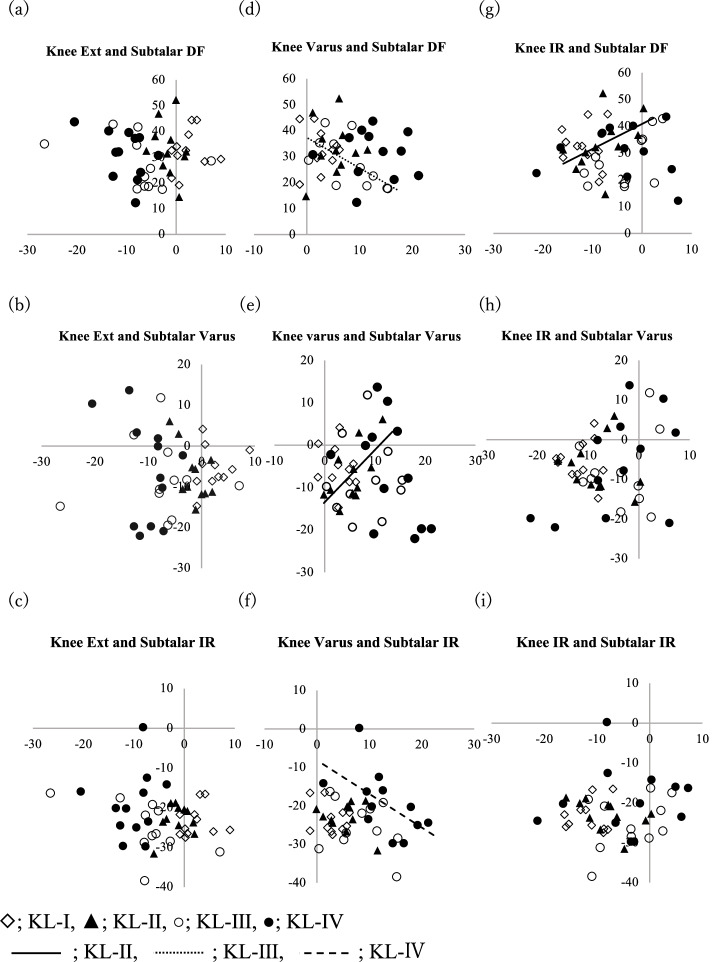
The relationship between the knee and subtalar joint is shown in a scatter plot. The horizontal axis is the knee joint angle, and the vertical axis is the subtalar joint angle (degree). Trendlines were filled in for items with significant differences between the knee and talocrural joint. Ext; Extension, DF; Dorsiflexion, IR; Internal Rotation

Table 4 and Fig. 4 (a-i) showed the correlation between knee joint angle and subtalar joint angle on each plane. There were no correlations between sagittal and axial plane joint angles between the knee joint and subtalar joint. On the coronal plane, significant correlations were found between knee joint varus angle and subtalar inversion angle in the limbs with KL-II knee OA (*r = 0.63, P < 0.05*) (Fig. 4-e). On the different plane, significant correlations were found between knee joint varus angle and subtalar dorsiflexion angle in the limbs with KL-III knee OA (*r = − 0.62, P < 0.05*) (Fig. 4-d), and knee joint varus angle and subtalar internal rotational angle in the limbs with KL-IV knee OA (*r = − 0.58, P < 0.0*5) (Fig. 4-f). As similar to the talocrural joint, significant correlations were found between knee joint tibial internal rotation angle and subtalar dorsiflexion angle in the limbs with KL-II knee OA (*r = 0.45, P < 0.05*) (Fig. 4-g).

**Table 4:** Correlation between the knee joint and subtalar joint in each grade of knee OA

Joint Plane			Correlation coefficient (*P* value)				
Knee	Subtalar	Chart	All	KL-I	KL-II	KL-III	KL-IV
Sagittal	Sagittal	a)	−0.073	0.218	−0.216	− 0.385	−0.430
			(0.633)	(0.520)	(0.523)	(0.243)	(0.163)
	Coronal	b)	−0.074	0.208	−0.591	0.026	−0.360
			(0.627)	(0.539)	(0.055)	(0.940)	(0.251)
	Axial	c)	−0.196	0.141	0.284	−0.552	0.149
			(0.198)	(0.680)	(0.398)	(0.078)	(0.644)
Coronal	Sagittal	d)	−0.191	−0.056	0.125	−0.629*	−0.034
			(0.208)	(0.871)	(0.713)	(0.038)	(0.917)
	Coronal	e)	−0.119	−0.220	0.634*	0.053	−0.491
			(0.436)	(0.515)	(0.036)	(0.878)	(0.105)
	Axial	f)	−0.153	0.006	− 0.147	0.129	0.580*
			(0.315)	(0.460)	(0.399)	(0.347)	(0.046)
Axial	Sagittal	g)	0.072	0.141	0.455*	−0.552	0.149
			(0.640)	(0.715)	(0.048)	(0.068)	(0.725)
	Coronal	h)	−0.997	−0.249	−0.283	− 0.314	0.580
			(0.211)	(0.987)	(0.665)	(0.280)	(0.091)
	Axial	i)	0.083	0.125	−0.491	0.387	0.043
			(0.588)	(0.714)	(0.125)	(0.239)	(0.895)

* Indicates significance at *P* < 0.05

Pearson’s correlation coefficients and Spearman’s rank correlation coefficients were used to determine the relationship between the alignment of the knee joint and the subtalar joint. In KL-II, the angle of the axial plane of the knee joint and the angle of the sagittal plane of the subtalar joint were correlated, and the angle of the coronal plane of the knee joint and the angle of the coronal plane of the subtalar joint were correlated. In KL-III, the angle of the coronal plane of the knee joint and the angle of the sagittal plane of the subtalar joint were correlated. KL-IV, the angle of the coronal plane of the knee joint and the angle of the axial plane of the subtalar joint were correlated. In KL-I, there was no correlation between knee and talocrural joints

## Discussion

The function of the hindfoot under weightbearing is known to compensate for the alignment of the knee joint, which is specifically compensated by the subtalar joint [22, 23]. In particular, calcaneal rotation had been shown to strongly relate to foot stability [24], and it is speculated that the subtalar joint complex, including the talocrural joint, affects a kinetic chain from the foot to the knee and hip joints, which influences the alignment of the lower extremities.

**Fig. 5:**
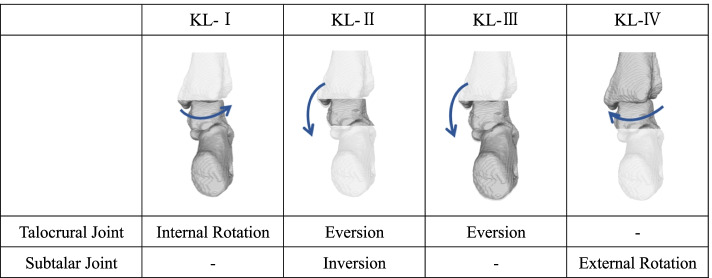
Correlation between knee varus and hindfoot alignment by grade of knee OA. The relationship between the motion of knee varus and the talocrural joint and subtalar joint in loading for each KL-grade is illustrated. KL-I, knee joint varus and talocrural joint internal rotation was correlated. KL-II, knee joint varus and talocrural joint eversion was correlated, and knee joint varus and subtalar joint inversion was correlated. KL-III, knee joint varus and talocrural joint eversion was correlated. KL-IV, knee joint varus and subtalar joint external rotation was correlated

Our findings from this study did not support our hypothesis that varus deformity of the knee joint causes changes in hindfoot alignment not only in the coronal plane but also in the sagittal and axial plane. However, when we looked at each grade of knee OA, it became clear that each grade had its own characteristic hindfoot alignment due to the effect of knee joint varus. In KL-I, the correlation was found with talocrural joint internal rotation, in KL-II, the correlation was found with talocrural joint eversion and subtalar joint inversion, in KL-III, the correlation was found with talocrural joint eversion, and in KL-IV, the correlation was found with subtalar external rotation (Fig. 5).

KL-I is characterized by minimal cartilage wear, good lubrication, and maintenance of tibial medial condyle movement comparing with normal knees. In the normal knee, with varus of the knee joint during loading, the tibial external rotation occurs as a kinetic chain, and it can be inferred that the same tendency was shown in KL-I. KL-II of our study showed the same trend as another study of alignment under full weightbearing condition [25]. This may be due to the possibility that eversion of the talocrural joint occurred by varus of the knee joint, while inversion of the subtalar joint occurred in order to keep stability of balance. But, in KL-III, compared to KL-I and KL-II, tibial external rotation angle of the lower leg was clearly increased. It was suggested that the subtalar joint may not be able to compensate for the balance of the alignment. Moreover, in KL-IV, there was no relationship between the knee joint and the talocrural and subtalar joints in the coronal plane, and there was a tendency for the subtalar joint to externally rotate. Similarly in KL-IV, it was suggested that the subtalar joint may not be able to compensate for the balance of alignment. Previous reports using only standing radiographs have shown that severe deformity of knee OA alignment disrupts the alignment of the ankle and increases the outward angle of the talocrural joint [26]. When the present study was classified according to KL-grade, there was no relationship between knee and talocrural joint angles on the coronal plane in KL-I without deformity or in KL-IV with significant deformity. This may be because the present study, unlike previous reports, assessed the knee joint angle in three dimensions. In the present study, the knee joint internal rotation angle was also related to the talocrural joint dorsiflexion angle, suggesting that there is a three-dimensional relationship. In the present study, tibial rotation of the knee joint also showed a marked increase in KL-IV. In KL-IV, not only changes in the alignment of the coronal plane of the knee joint, but also changes in the horizontal or sagittal plane may affect the ankle joint. In fact, according to recent research report, KL-IV causes not only simple joint misalignment but also anterior-posterior degeneration of the meniscus in the knee joint, which is rarely seen in KL-III [27]. In other words, this suggests that in KL-IV, the tibial rotation of the knee joint might have various effects on the alignment of the lower extremities.

According to recent reports, knee OA progresses with rotation of the femur, and the degeneration progresses rapidly from KL-III [28]. In this study, there was no significant difference in the angle of rotation of the knee joint between KL- I and II, suggesting that the patients did not require compensation for the degeneration of knee joint alignment. On the other hand, in KL-IV, the degree of knee joint deformity was so great that it was difficult to achieve sufficient postural control by compensating with the ankle joint. From the viewpoint of postural control, it is known that the knee adduction moment of the knee joint increases in severe cases of knee OA, especially during walking [29], and compensation for postural maintenance in the frontal plane is considered to be significant.

Although the relationship between the knee joint and the hindfoot was found only on the axial plane, this study is considered to be useful in the following two points. The first is the usefulness of the upright CT used in this study. Although Kaneda et al. [25] and Ota et al. [30] have described the assessment of lower limb alignment under load, the present study is novel in that it comprehensively assesses the alignment of multiple large joints under load. Secondly, it includes new insights into how to approach patients with knee OA in nonoperative intervention. Although the external wedge is mainly involved in controlling the anterior surface movement and has been used as an orthotic treatment for pain relief in knee OA, future studies on insoles that take into account not only the anterior surface movement but also the rotation movement for hindfoot alignment may contribute to the development of innovative insoles that are optimal for each knee OA case.

One of the limitations of this study is that the assessment of lower extremity alignment is limited to a static standing position. Pain in patients with knee OA is often due to movements such as walking, and a detailed comparison of alignment at rest and in motion is necessary. In particular, recent reports have shown that the standing alignment of knee OA is strongly related to the knee adduction moment during walking [31], and the relationship between the assessment of lower limb alignment during standing and dynamic assessment needs to be investigated. Secondly, the alignment of the knee joint is at least influenced by the alignment of the hip joint as well as the ankle joint [32]. In this study, subjects were recruited from patients who came to the hospital for treatment of knee OA. Therefore, data from healthy subjects were not included in the study, which is a subject for future research. The present study did not examine the relationship between hip and knee joint angle, which needs to be examined in the future. In addition, the evaluation of lower limb alignment under different loading conditions, such as when wearing shoes or full load, is another issue to be addressed in the future. Finally, the present study included only 45 limbs of 25 knee OA patients which has limited statistical power. In addition, it includes a large number of subjects with two knee joint measurements in one patient. Knee OA is a bilaterally symptomatic disease [33], and the number of cases needs to be increased to examine the association in both unilateral and bilateral conditions. However, our results clearly describe the difference of 3D alignment of the knee, talocrural and subtalar joints in each knee OA grade during standing.

## Conclusion

The results of this study showed that the varus and valgus knee joint angles in KL-II and III were related to the varus and valgus ankle joint angles, but not to other joint angles. The varus and valgus knee joint angles in KL-II was also related to the varus and valgus subtalar joint angles. As knee OA worsened, it became apparent that compensatory knee joint alignment depended on the ankle joint rather than the subtalar joint. It is difficult to elucidate the alignment of the ankle joint under loading in more detail without standing CT, and the results of this study are significant in clarifying the characteristics of lower limb alignment under load in more detail in cases of knee OA.

## Data Availability

The datasets of the present study are available from the corresponding author upon reasonable request.

## Abbreviations

OA: Osteoarthritis
3D: Three-dimensional
2D: Three-dimensional
CT: Computed tomography
KL: Kellgren-Lawrence
DICOM: Digital Imaging and Communication in Medicine
FTA: Femorotibial angle
BMI: Body mass index
